# Structure of eukaryotic purine/H^+^ symporter UapA suggests a role for homodimerization in transport activity

**DOI:** 10.1038/ncomms11336

**Published:** 2016-04-18

**Authors:** Yilmaz Alguel, Sotiris Amillis, James Leung, George Lambrinidis, Stefano Capaldi, Nicola J. Scull, Gregory Craven, So Iwata, Alan Armstrong, Emmanuel Mikros, George Diallinas, Alexander D. Cameron, Bernadette Byrne

**Affiliations:** 1 Department of Life Sciences, Imperial College London, London SW7 2AZ, UK; 2 Faculty of Biology, University of Athens, Panepistimioupolis, 15781, Athens, Greece; 3 Faculty of Pharmacy, University of Athens, Panepistimioupolis, 15771 Athens, Greece; 4 Department of Biotechnology, University of Verona, Strada Le Grazie 15, 37134 Verona, Italy; 5 Department of Chemistry, Imperial College London, London SW7 2AZ, UK; 6 JST, Research Acceleration Program, Membrane Protein Crystallography Project, Konoe-cho, Yoshida, Sakyo-ku, Kyoto 606-8501, Japan; 7 School of Life Sciences, University of Warwick, Gibbet Hill Road, Coventry CV4 7AL, UK

## Abstract

The uric acid/xanthine H^+^ symporter, UapA, is a high-affinity purine transporter from the filamentous fungus *Aspergillus nidulans*. Here we present the crystal structure of a genetically stabilized version of UapA (UapA-G411V_Δ1–11_) in complex with xanthine. UapA is formed from two domains, a core domain and a gate domain, similar to the previously solved uracil transporter UraA, which belongs to the same family. The structure shows UapA in an inward-facing conformation with xanthine bound to residues in the core domain. Unlike UraA, which was observed to be a monomer, UapA forms a dimer in the crystals with dimer interactions formed exclusively through the gate domain. Analysis of dominant negative mutants is consistent with dimerization playing a key role in transport. We postulate that UapA uses an elevator transport mechanism likely to be shared with other structurally homologous transporters including anion exchangers and prestin.

## Abstract (web summary)

UapA is a uric acid/xanthine H^+^ symporter from a filamentous fungus. Here, the authors solve the crystal structure of the transporter in complex with xanthine revealing it to be a dimer, and this homodimerisation is proposed to be important for function.

## Graphical abstract

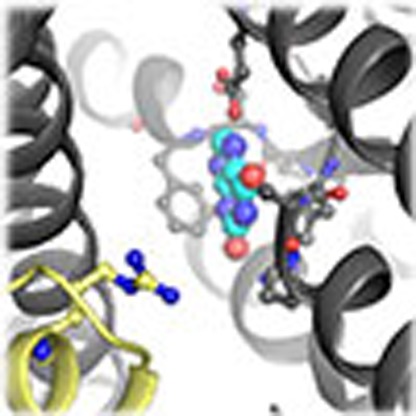

Transporters have evolved to specifically carry selected molecules across membranes. Nucleobase–ascorbate transporters (NATs), found in almost all species from bacteria and fungi to plants and mammals, are responsible for cellular uptake of essential metabolites^1^,^2^. In bacteria, plants and fungi, NATs are specific for nucleobases, while human members of this family transport ascorbate (vitamin C)^3^. NAT transporters also have the capacity to mediate the uptake of toxic purine analogues^4^,^5^. UapA from *Aspergillus nidulans* is specific for the purines xanthine and uric acid but does not transport hypoxanthine or adenine^6^. UapA is an intensively studied transporter due to the development of simple genetic, biochemical and cellular assays, possible in a model microbial eukaryote, such as *A. nidulans*^2^. Such experiments have allowed us, not only to gain an insight into the residues critical for xanthine binding and transport by UapA, but importantly to also isolate randomly generated mutations that change the specificity of the protein and enable non-native substrates to be transported^7^,^8^,^9^. In general, these mutations do not occur in the binding site but rather in positions that could not have been predicted *a priori*. In one experiment, 20 out of the 28 unbiased mutations that enabled the fungus to use hypoxanthine were observed to involve mutations of Arg481 (ref. 8). The structure of the *Escherichia coli* homologue, UraA^10^, however, failed to provide an adequate explanation for this, or indeed for other mutations that affect specificity.

UapA has previously been shown to exist as dimers in the membrane and in detergent-based solution^11^. As with other transporters^12^, there is evidence that UapA oligomerization is important for trafficking of UapA to the plasma membrane^11^. Oligomerization is suggested to have a potential role in the function of other transporters, however as yet these observations lack a detailed structural context^13^,^14^,^15^,^16^. It is currently unclear whether dimerization has a functional role in UapA.

Here we present the structure of UapA, which has allowed us to put the large body of genetic studies in a structural context and provide evidence for dimerization playing a key role in transporter function.

## Results

### Overall structure of UapA reveals homodimer formation

A thermostabilized construct of UapA with a single-residue substitution, G411V, and lacking the N-terminal 11 residues (UapA-G411V_Δ1–11_)^17^ was expressed in *Saccharomyces cerevisiae* as a green fluorescent protein (GFP) fusion protein^18^. UapA-G411V_Δ1–11_ binds xanthine but is transport inactive^17^,^19^. The structure in complex with xanthine was solved by single isomorphous replacement with anomalous scattering and refined at a resolution of 3.7 Å to an *R*-factor of 29.6% and *R*_free_ of 32.7% (Supplementary Table 1).

**Figure 1:**
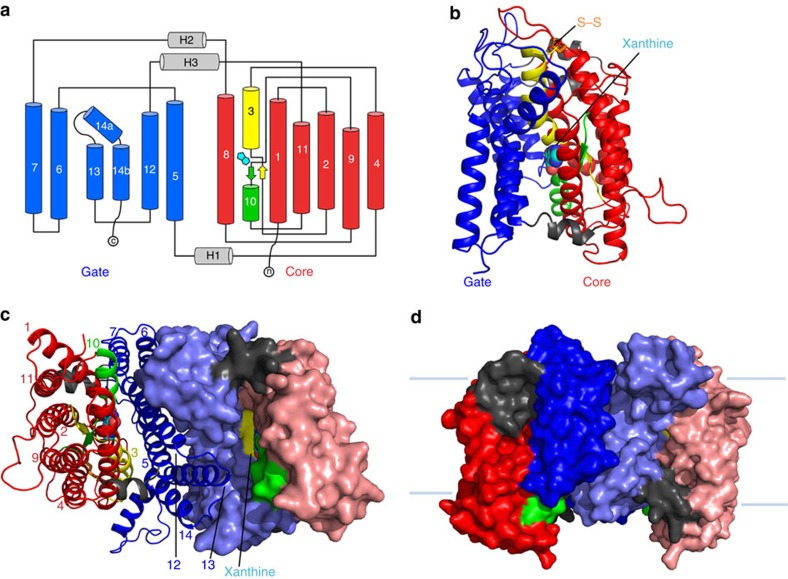
Structure of UapA. (**a**) Topology diagram of UapA. α-helices are represented by cylinders and β-strands by arrows. The substrate-binding site between the amino termini of TMs 3 and 10 is indicated by the schematic of xanthine (cyan). The protein is arranged into two domains, a gate domain consisting of TMs 5, 6, 7, 12, 13 and 14 (blue), and a core domain consisting of TMs 1, 2, 3, 4, 8, 9, 10 and 11 (red). The half helices and short β-strand regions of TMs 3 and 10 (both part of the core domain) are coloured yellow and green, respectively. (**b**) Ribbon representation of the UapA monomer. The gate domain is shown in blue while most of the core domain is shown in red. The amphipathic helices that link the core and gate domains are shown in grey. Xanthine is shown in cyan as a space-filling model and the disulphide bond is shown in orange sticks. Only the N-terminal (residues 12–65) and C-terminal (residues 546–574) ends are missing from the structure. (**c**) UapA dimer from the cytoplasmic side. One monomer is shown as in **b**, with the helices numbered, and the other is shown in surface representation with the same colouring as in **a**. The xanthine in the surface representation is labelled. (**d**) Surface representation of the dimer looking through the membrane. The pale blue lines indicate the likely location of the membrane.

The overall structure of UapA contains 14 transmembrane domains (TMs) organized into a 7+7 TM fold divided into a core (TMs 1–4 and 8–11) and a gate (TMs 5–7 and 12–14) domain, similar to UraA^10^ (Fig. 1a,b and Supplementary Fig. 1). A key feature is that TMs 3 and 10 only extend halfway through the protein, being followed by short β-strands and random coils that crossover in the centre of the protein (Fig. 1a,b). Compared with the bacterial protein UraA, UapA contains substantially longer loop regions (Supplementary Fig. 2). Interestingly, the extracellular loop between TMs 3 and 4 contains a disulphide bond between Cys174 and Cys185, residues highly conserved in fungi (Fig. 1b and Supplementary Fig. 3). Mutating either residue to serine results in both marked reduction in heterologous expression and almost complete abolition of sorting to the membrane (Supplementary Fig. 3) indicating that the formation of this disulphide bond is important for correct intracellular folding and localization of UapA. This is also the case for other eukaryotic membrane transport proteins^20^,^21^. UapA forms a dimer with an extensive interface and a buried surface area of 6,000 Å^2^, involving TMs 12, 13 and 14 of the gate domain. TM 13 is particularly closely associated with dimer formation, fitting into a cleft formed by the opposite monomer (Fig. 1c,d).

### The UapA substrate-binding site

**Figure 2:**
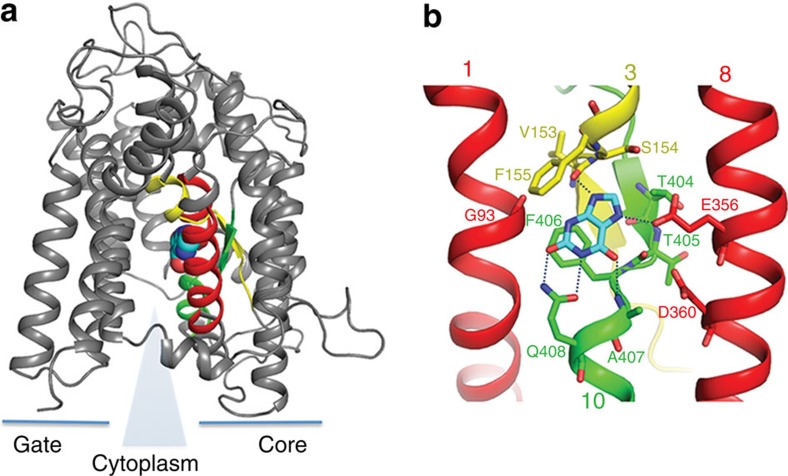
Substrate-binding site and specificity regions of UapA. (**a**) A monomer of UapA shown in grey with the key substrate-binding regions of the protein coloured as in Fig. 1. The blue triangle indicates the solvent-filled translocation channel from the substrate-binding site to the cytoplasm. Xanthine is shown in cyan space-filling model. (**b**) Zoomed-in version of the xanthine-binding site coloured as in Fig. 1 and with xanthine shown in stick model. Residues involved in substrate binding are shown in stick representation and labelled. Side chain and main chain groups of Glu356, Gln408 and Ala407 and main chain groups of Phe155, Val153 are all within H-bonding distance of the xanthine. Essentially the main contacts are between the amide N of Phe155 and N9 of xanthine, Glu356 and N7–H, Gln408 and N1–H and C2=O, and the amide N of Ala407 and C6=O. The side chain groups of Phe155 and Phe406 also pack around the xanthine. Other residues from TMs 1, 3 and 8 contribute to the architecture of the binding site as indicated.

Density consistent with xanthine is visible in a clearly defined binding site approximately halfway across the membrane in a cleft formed by the half helices of TMs 3 and 10 (Fig. 2a; and extended data, Fig. 4), similar to the uracil-binding site in UraA. A number of residues were predicted to be involved in xanthine binding by previous mutational analysis^7^,^8^,^19^. The current structure confirms many of these earlier predictions, revealing xanthine is within hydrogen-bonding distance of the side chains of Gln408 (TM 10) and Glu356 (TM 8), the main chain nitrogen of Ala407 at the N terminus of TM 10 and the carbonyl oxygen of Val153 preceding TM 3, as well as the main chain nitrogen of Phe155 of TM 3. The phenyl rings of both Phe155 and Phe406 pack onto the xanthine (Fig. 2b). The orientation of the substrate (Fig. 2b) is corroborated by the position of the electron density peak in an anomalous difference map obtained for a complex of UapA crystallized with 8-bromoxanthine (Supplementary Fig. 4), and with structure activity relationships of xanthine analogues indicating Q408 interacts with N1–H and C2=O of xanthine while E356 interacts with N7–H (ref. 9). With the current structure we cannot completely rule out the substrate adopting the alternative conformation with E356 interacting with N9–H, however in light of all the current evidence the most likely orientation of xanthine is the one presented in Fig. 2b. The residues directly interacting with the xanthine are all from the core domain and in the structure a detergent molecule packs over the xanthine separating it from TM 12 of the gate domain (Supplementary Fig. 5). The xanthine is fully accessible to the inward-facing side of the protein through this detergent-filled cavity.

### The neighbouring subunit modulates substrate specificity

**Figure 3:**
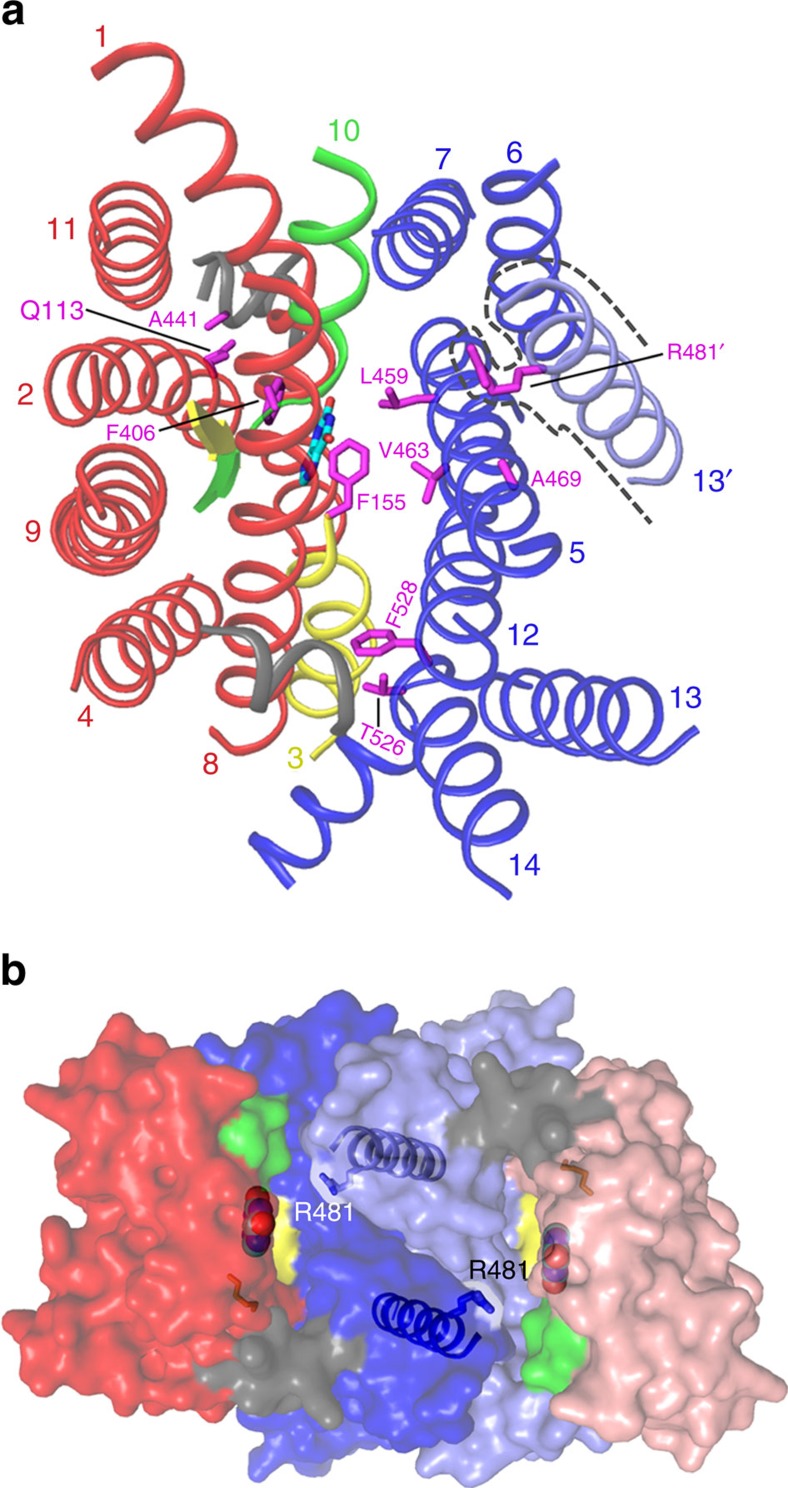
UapA substrate specificity. (**a**) Location of the different residues involved in substrate specificity. The helical regions of the protein are shown as cylinders coloured as in Fig. 1. TM 13 from the opposite monomer is shown in light blue and indicated by the black dotted line, which traces around the surface of the TM. For clarity the majority of the loop regions of UapA have been removed. The individual residues involved in substrate specificity are shown in magenta stick model and labelled. Xanthine is shown in cyan stick model. (**b**) Structure of the UapA dimer as a surface representation showing the close proximity of R481 from the opposing monomer, to the substrate-binding site. TM 13 in both monomers is shown in ribbon representation, with R481 indicated in stick representation. Xanthine is shown with cyan carbon atoms.

Wild-type (WT) UapA cannot bind hypoxanthine or adenine (Supplementary Fig. 6). However, mutants allowing uptake of these purines have been isolated through unbiased genetic approaches^22^. Mutants selected from these screens are shown in Supplementary Fig. 6. The residues affected primarily fall into one of three different regions of the protein. Phe406 (ref. 8) is located within the binding site. Val463, Ala469 (TM 12)^8^, Thr526 (ref. 7) and Phe528 (TM 14)^22^ are clustered at the interface between the gate and core domains near to the substrate-binding site (Fig. 3a). Gln113 and Ala441 (refs 7, 8) are located distant from the binding site in putative hinge regions between the two domains (Fig. 3a). In addition, specificity mutations of Arg481 arise often in the genetic screens^8^. These mutations do not affect transport of physiological substrates but rather modulate the specificity of the protein allowing low-affinity transport of hypoxanthine or adenine. At first glance and considering the monomer in isolation, the role of Arg481 in substrate specificity is difficult to interpret. However, the UapA dimer structure reveals that this residue lies in close proximity to the xanthine-binding site, but of the opposite monomer (Fig. 3b). MD simulations, in fact, suggest that the bound xanthine and Arg481 come in much closer proximity (2 Å) than observed in the crystal structure (12 Å) (Supplementary Figs 7 and 8).

The most efficient transport of hypoxanthine and adenine is observed by combining mutations from the different regions described above (Supplementary Fig. 6). Mutations within the binding site, such as Q408E, which allows efficient binding (*K*_i_=71 μΜ) but no transport of hypoxanthine, or F406Y, which permits only very low transport activity (>2 mM) for hypoxanthine, when combined with mutations outside the binding site (Q113L, A469E, T526M or F528S) convert UapA into a very efficient hypoxanthine and adenine transporter (Supplementary Fig. 6). Similarly, combinations of mutations in distinct specificity regions (Q113L/F528S, A441V/F528S, V463I/F528S, A469E/F528S, R481G/F528S, R481G/T526M, Q408E/R481G/F528S and Q408E/R481G/T526M) also result in an expanded substrate profile of UapA (Supplementary Fig. 6). From the structure, we predicted that Phe155 and Leu459, both involved in substrate co-ordination, might also have roles in substrate specificity and indeed mutations of these residues confer low-affinity binding of hypoxanthine and adenine (Supplementary Fig. 6).

### Evidence that dimerization is required for function

**Figure 4:**
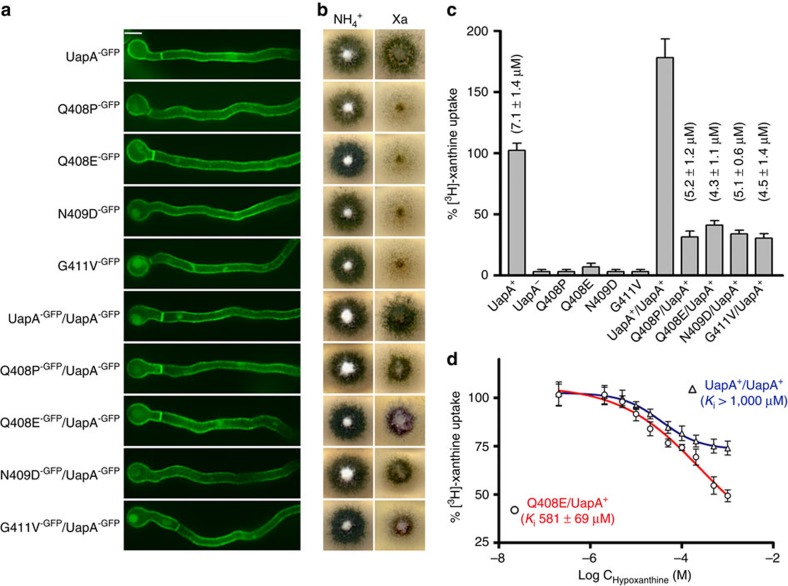
Importance of dimer formation for UapA transport function. (**a**), *In vivo* inverted fluorescence microscopy of growing hyphal cells of isogenic strains expressing GFP-tagged WT UapA or mutant versions Q408P, Q408E, N409D or G411V, or co-expressing two copies of UapA (UapA/UapA) or a copy of UapA with each one of the four mutants (Q408P/UapA, Q408E/UapA, N409D/UapA or G411V/UapA). In all cases UapA-GFP fluorescence is associated with the plasma membrane and the septa of growing hyphal cells, a picture typical of properly folded and correctly localized UapA^7^,^8^. Minor cytoplasmic GFP labelling reflects the normal vacuolar turnover of UapA^56^. Scale bar shown in the UapA-GFP panel, 5 μm. (**b**) Relevant growth tests of the same strains on either ammonium (NH_4_^+^) or xanthine (Xa) as sole nitrogen sources. Notice that full growth on Xa occurs in UapA or UapA/UapA strains, whereas co-expression of the selected loss-of-function mutants with WT UapA (last four subpanels) reduces growth. (**c**) Initial uptake rates of [^3^H]-xanthine in strains expressing WT or mutant versions of UapA. Rates of WT UapA are taken as 100%. Measurable *K*_m_ values for xanthine are also indicated. Error bars represent s.d., *n*=9. (**d**) The effect of hypoxanthine on [^3^H]-xanthine uptake on strains expressing WT UapA (blue line) or co-expressing Q408E/UapA (red line) was assessed. Rates of xanthine uptake in the absence of hypoxanthine for both strains are taken as 100%. *K*_i_ values are also indicated. Error bars represent s.d., *n*=9. Note that solubility issues mean it is not possible to use higher concentrations of hypoxanthine.

Recent genetic, cellular and biochemical studies demonstrated that dimerization is critical for UapA trafficking and turnover^11^. However, the observed UapA dimer and the effect of Arg481 on specificity prompted us to explore whether dimerization also has a role in substrate transport. A second copy of UapA can be co-expressed, through plasmid integration, together with the endogenous copy, so that the overall UapA activity is roughly doubled (Fig. 4c). We carried out co-expression experiments of the endogenous WT UapA with each one of four kinetically distinct UapA mutants exhibiting undetectable (G411V, Q408P and N409D) or significantly reduced (Q408E) transport activities, when expressed in a purine uptake-deficient strain. All mutants showed similar protein expression levels to the WT^7^ and normal localization into the plasma membrane (Fig. 4a). G411V and N409D are capable of substrate binding, similar to the WT, but lack detectable transport activity. Q408P has >100-fold reduced xanthine-binding affinity (*K*_m_>1 mM) and consequently also lacks detectable transport activity at low substrate concentrations. Q408E has reduced transport activity (5–10% of the WT), a nearly normal *K*_m_ of xanthine or uric acid, but relatively high-affinity binding (71 μΜ) for hypoxanthine, which however cannot be transported (Fig. 4a). WT UapA has a *K*_m_ of 7.0±2.0 μΜ (±indicates s.d., *n*=9) for xanthine^7^,^19^. All mutants when co-expressed with WT had a dominant negative effect for growth on xanthine (Fig. 4b) and reduced [^3^H] xanthine uptake (28–41% of WT), but had little effect on the *K*_m_ value for xanthine (4.3–5.2±1.4 μΜ, ±indicates s.d., *n*=9; Fig. 4c). This strongly suggests that in all cases the ‘heterodimeric' complexes exhibited low levels of transporter function, so that the transport rates measured reflect mostly, if not entirely, the WT/WT UapA dimers. As the G411V construct, for which the structure was solved, was included in these experiments it seems unlikely that this dominant negative effect could be due to effects of the mutations on protein folding. Most importantly in the Q408E/WT strain, UapA-mediated xanthine transport could be significantly inhibited by hypoxanthine, in contrast to the WT control. This is clearest at 0.5 mM hypoxanthine, where inhibition in Q408E/WT is 48% but only 21% for WT (Fig. 4d).

### Molecular dynamics support a role for Arg481 in the dimer

**Figure 5:**
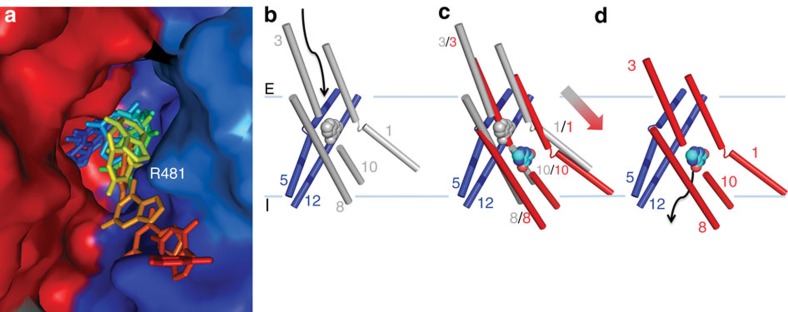
Substrate translocation pathway and transport mechanism in UapA. (**a**) Surface model of the UapA dimer showing the xanthine trajectory during MD. The image is zoomed into the substrate translocation channel. The regions of the protein dimer are coloured as in Fig. 1, and R481 from the opposite monomer is labelled. The positions adopted by the xanthine during translocation (Supplementary Figs 7 and 8) are coloured from the xanthine-binding site (blue) to the intracellular side of the protein (red). (**b**–**d**) UapA is postulated to function using an elevator mechanism, involving displacement of TMs 1, 3, 8 and 10 of the core domain, which effectively carries the substrate from one side of the membrane to the other. (**b**) Postulated outward-facing structure of key regions of UapA, with the helices shown as cylinders. The positions of grey-coloured helices (TMs 1, 3, 8 and 10 from the core domain) are modelled based on the outward-facing structure of AE1 (PDB 4YZF). The positions of helices 5 and 12 (blue) are as in the crystal structure. The arrow indicates access to the postulated substrate-binding site shown by the grey space-filling model of xanthine. (**c**) Overlay of the helices 1, 3, 8 and 10 in the postulated outward-facing conformation (grey) and the inward-facing conformation from the crystal structure (red). The position of xanthine in both conformations is shown in grey (outward facing) and cyan (inward facing). The large grey–red-coloured arrow indicates the direction of movement of the core domain helices during change from the outward- to inward-facing conformations. (**d**) Inward-facing conformation of the helices in blue and red as seen in the crystal structure. Xanthine is shown in cyan. The large arrow indicates the substrate translocation trajectory from the binding site to the intracellular side of the membrane (as seen in **a**). Although this seems to be the key conformational change it is possible that other domain movements are involved, for example, TM 14 is kinked and this may need to flex for the core to move. Interestingly Thr526 and Phe528, key for specificity, are located at this kink.

Molecular dynamics simulations suggest a translocation pathway from the binding site to the inside of the cell (Fig. 5a and Supplementary Figs 7 and 8). Given the resolution of the crystal structure, these data should be treated with caution; however, the results are consistent with the biochemical data. Initially, xanthine remains strongly ligated to Gln408 whilst tumbling within the binding cavity. It interacts with Leu459, Val463 and Ser466 close to the binding site while Arg481 of the opposite monomer approaches the central binding cavity. This effectively creates a specificity barrier on the pathway to the cytoplasm with the xanthine forming transient H-bond and π–π stacking interactions with Arg481 before finally moving to the cytosol with characteristic conformational changes of the Arg481 side chain and the Arg481–Gln408 interaction (Fig. 5a and Supplementary Fig. 8). During this procedure there is a gradual displacement of TM 10, which may be required for ligand exit.

### UapA is likely to function by an elevator mechanism

Comparison of the UapA structure with other transporters provides additional insight into the translocation pathway and transport cycle. Recently, structures have been determined of members of the SLC26 (ref. 23) and SLC4 (ref. 24) transporter families (Supplementary Fig. 9), showing them to be structurally homologous to the NAT family. Taken individually, the core and gate domains of UapA, UraA, anion exchanger 1 (AE1) and SLC26Dg superimpose remarkably well, given their low sequence homology. The largest outlier is the gate domain of UraA where TMs 6 and 7 adopt a different position with respect to the two central TMs of the domain (TMs 5 and 12). Overall, the major differences between the proteins are the positions of the core relative to the gate domain and, as has been reported previously, transport is likely to be effected by the movement of one domain against the other. Whereas UapA, UraA and SLC26Dg are all inward facing, AE1 is outward facing. Arakawa *et al.*^24^ proposed a mechanism based on the inward-facing UraA structure and the outward-facing AE1 structure, although this was limited by the substantially different gate domains in the two proteins. The structure of UapA allows us to refine the model. For UapA the tight interaction between the gate domains at the dimer interface suggests that this would remain relatively static and this is reflected in the distribution of the temperature factors (Supplementary Fig. 10). If we superpose all the structures by their respective gate domains we see that the core domain of AE1 is displaced along the trajectory of TMs 1, 3, 8 and 10 by 10 Å compared with the equivalent domain in the inward-facing structures. Since the ligand in UapA is entirely located within the core domain such a movement would effectively carry the substrate across the membrane (Fig. 5b–d). Such an elevator mechanism, first described for Gltph^25^, has now been reported for a number of different proteins^26^,^27^ including the sodium-proton antiporters, which also contain two domains with the putative substrate-binding site at the interface of two unwound helices in the core domain^28^,^29^. This mechanism would be consistent with the reported loss of activity when Gly411 is replaced with the bulkier valine as in the crystallized G411V_Δ1–11_ construct. The mutation clearly does not prevent xanthine binding, and comparing TM 10 with the equivalent helix in UraA does not indicate that it has a major effect on the local conformation of the protein. It could instead sterically hinder the core domain from gliding over the residues of the gate domain. A replacement of Gly411 with Leu is also inactive, whereas the less bulky Pro or Ala substitutions at this site retain activity^19^.

## Discussion

UapA, like many other membrane transport proteins, is dimeric. This was previously shown to be important for correct trafficking of the protein to the plasma membrane^11^. Our results suggest, however, that dimer formation is also important for both specificity and transport. First, the crystal structure shows that the N terminus of TM 13 of one subunit, including the side chain of Arg481, contributes to the inward-facing translocation channel of the opposing subunit. It is interesting to note that Arg481 is the most commonly mutated residue in genetic selection experiments designed to alter the specificity of the protein to allow transport of the non-native substrate, hypoxanthine^8^. Indeed, it is possible that Arg481 from the opposite monomer acts as one of a number of checkpoints ensuring transport of the correct substrate. Second, dominant negative mutations indicate that one subunit influences the transport function of the other. The exact structural mechanism for this dominant negative effect is more difficult to pin down. There are a number of pointers towards an elevator-type mechanism for this protein including the fact that the xanthine in the structure exclusively interacts with residues of the core domain. In addition, comparison of the inward-facing UapA and the structurally homologous outward-facing AE1 (ref. 24) reveals a marked shift in the position of the core domain relative to the gate domain. As an extension of this reasoning, the structurally related human anion exchangers and prestin^23^,^24^ may also use the elevator mechanism. Further work will be required to provide conclusive evidence. The question remains, however, how a simple elevator mechanism would be consistent with the dominant negative effect we observe for UapA. For the SWEET transporters, which form trimers and most likely use a rocker switch mechanism, dominant negative effects between subunits were observed via changes in the cytoplasmic gate^16^,^30^. Almost all the UapA mutations identified in the unbiased genetic screen that allow the protein to transport the non-native substrates, hypoxanthine and adenine (Supplementary Fig. 6), are seen either at the interface between the core and gate domains or in the linker between the two domains. These may have subtle effects on transport by changing the relative positions of the gate and core domains. Other conformational changes in addition to the elevator movement are likely to be required during transport, as seen for other proteins^31^. These may involve coupling between the subunits of UapA, accounting for the dominant negative effect. Our results however provide evidence for UapA dimerization playing a role in fine-tuning the substrate selectivity process.

## Methods

### Expression and isolation of UapAG411V_Δ1–11_

The UapAG411V_Δ1–11_ construct was generated using the QuikChange mutagenesis kit (Stratagene), and PCR followed by cloning by homologous recombination into pDDGFP^17^. The sequences of the oligonucleotide primers used for both mutagenesis and cloning are provided in Supplementary Table 2. The expressed protein construct incorporates a C-terminal tobacco etch virus cleavage site followed by a GFP-His8 tag^32^. The UapAG411V_Δ1–11_ was transformed into the *S. cerevisiae* FGY217 strain^33^ and positive transformants selected using -URA plates. Selected transformants were grown in -URA medium with 0.1% glucose at 30 °C, with shaking. Protein expression was induced with a final concentration of 2% galactose. After 22 h of induction, the cells were collected by centrifugation before resuspension in cold cell resuspension buffer (50 mM Tris-HCl (pH 7.6), 1 mM EDTA and 0.6 M sorbitol) supplemented with protease inhibitors (Roche). Cells were lysed using a cell disruptor (Constant Systems) at 4–10 °C. Unbroken cells and debris were removed by centrifugation at 15,000*g* and 4 °C for 10 min and the membranes isolated by further centrifugation at 150,000*g* at 4 °C for 60 min to isolate the membranes. The supernatant was discarded and the membrane pellet resuspended in membrane resuspension buffer (20 mM Tris-HCl (pH 7.5) and 0.3 M sucrose) to a final volume of 6 ml l^−1^ starting cell culture using a handheld glass homogenizer (Kimble Chase). The membranes were solubilized in membrane solubilization buffer (1 × PBS, 150 mM NaCl, 10% (v/v) glycerol, 1% (w/v) DDM_LA_ (Generon) and protease inhibitors) supplemented with 1 mM xanthine (Sigma) or 8-bromoxanthine (see below for details of the preparation) with constant stirring at 4 °C for 1 h. The sample was centrifuged (100,000*g* for 45 min at 4 °C) to remove insoluble material, and imidazole at a final concentration of 10 mM was added to the detergent-solublized material. The soluble protein was mixed with 20 ml of Ni^2+^-NTA superflow resin (Qiagen) pre-equilibrated with buffer A (1 × PBS, 150 mM NaCl, 10 mM imidazole (pH 7.5), 1 mM xanthine or 8-bromoxanthine, and 0.03% (w/v) DDM_LA_) at 4 °C for 2 h. The resin was then packed into a glass econo-column (Bio-Rad). Following extensive washing with buffer A supplemented with 10 and then 30 mM imidazole, bound protein was eluted with five column volumes of buffer B (20 mM Tris-HCl (pH 7.5), 150 mM NaCl, 1 mM xanthine or 8-bromoxanthine, and 0.03% (w/v) DDM_LA_) supplemented with 250 mM imidazole. The GFP tag was removed from the fusion protein by treatment with an equimolar concentration of His-tagged tobacco etch virus (TEV) protease. The protein sample was then dialysed overnight in buffer B supplemented with 10% glycerol using 12-kDa molecular weight cutoff dialysis tubing (Spectrum Labs). The remaining protein sample was passed through a 0.22-μm filter (Millex) to remove any precipitation before loading onto a 5-ml His-trap column (GE Biosciences). The His-tagged GFP and TEV protease bind to the column, while the UapAG411V_Δ1–11_ is found in the flow-through. The UapAG411V_Δ1–11_ was concentrated to 0.5 ml in 100-kDa molecular weight cutoff filters (Millipore). Aggregates were removed from the sample by centrifugation at 18,000*g* and 4 °C for 10 min. The sample was then loaded onto a Superdex 200 10/300 gel filtration column equilibrated with 20 mM Tris (pH 7.5), 150 mM NaCl, 0.6 mM xanthine or 8-bromoxanthine, and 0.03% DDM_LA_ (ref. 17). The peak fractions were concentrated to 12 mg ml^−1^ using a 100-kDa molecular weight cutoff filter (Millipore) and either used immediately for crystallization trials or stored at −80 °C.

### Localization analysis of the C174S and C185S mutants

The C174S and C185S mutants were generated (for mutagenic primer sequences see Supplementary Table 2) in the UapAG411V_Δ1–11_ background and expressed as described above. FGY217 cells expressing either the UapAG411V_Δ1–11_ or the C174S or C185S mutants (in the UapAG411V_Δ1–11_ background) as C-terminal GFP fusion proteins were collected by centrifugation and the expression level (mg l^−1^) of the different constructs assessed using GFP fluorescence based on a standard curve of known GFP concentrations and taking into account the intrinsic fluorescence (3,000 relative fluorescence units) of uninduced FGY217 cells^32^. Two hundred-microlitre aliquots of cells were fixed in paraformaldehyde and then washed and resuspended in KPO_4_/sorbitol before visualization using a Zeiss LSM-510 inverted confocal microscope fitted with a Plan-Apochromat × 63 objective.

### Crystallization and structure determination

UapAG411V_Δ1–11_ crystals were initially obtained using the MemSys/MemStart screen (Molecular Dimensions) in vapour diffusion sitting drop plates. Optimized crystals were obtained in 0.1% MES (pH 6.5), 30% PEG300, 0.03% DDM_LA_, 1% *n*-hexyl-β-D-glucopyranoside vapour diffusion sitting drops at 20 °C. Crystals appeared overnight and were frozen immediately in liquid nitrogen for storage and transport to the synchrotron. Data were collected at Diamond Light Source, UK. The crystals always showed anisotropic diffraction, with the best crystals diffracting X-rays to Bragg spacings of 3.5 Å in the strongest direction. Initial phases were calculated by single isomorphous replacement with anomalous scattering using a crystal soaked overnight with saturating concentrations of TaBr and an isomorphous crystal that was treated with 10 mM TaBr but showed no TaBr occupancy.

Diffraction images were processed using XDS^34^ through the Xia2 pipeline^35^ and the CCP^36^ suite of programmes. The space group was determined to be P2_1_ with two molecules in the asymmetric unit. The heavy atom coordinates were determined using SHELX^37^. Their positions were refined and phases calculated with SHARP^38^. The maps were improved by averaging over the two protomers of the asymmetric unit, including the high-resolution data set as a second crystal in dmmulti^39^. Initial maps showed clearly the positions of the transmembrane helices and many of the linking loops. After building into these maps using Coot^40^ and O (ref. 41) a model was obtained. Structural refinement was performed using PHENIX^42^ initially against data that had been anisotropy-corrected using the diffraction anisotropy server. In the first cycle DEN^43^ refinement as implemented in Phenix was carried out. Though this distorted the model it also resulted in improved maps, which after averaging using RAVE^44^ enabled a better model to be built, as judged by the behaviour in subsequent refinement cycles. Refinement was carried out with the application of non-crystallographic symmetry and secondary structure restraints with grouped B-factors and each of the two molecules of the asymmetric unit assigned to a separate TLS group^45^. The model was improved by interactive model building and refinement with maps improved by NCS averaging and B-factor sharpening^46^. Regular refinement in Phenix was interspersed with Rosetta refinement^47^ again as implemented in Phenix. This gave significantly improved geometry and lower *R*-factors in subsequent cycles. In general the density was better for molecule A where continuous density was obvious for all of the loop regions enabling residues to be tentatively, given the low resolution, placed (Supplementary Fig. 11). The residues that are outliers in the Ramachandran plot^48^ are all in these loop regions. In the final structure monomer A contains all residues from 66 to 545 while residues 133–144 are missing for monomer B. Xanthine was placed in clear density between TMs 3 and 10. Density consistent with detergent was also observed near to the substrate-binding site. This has been modelled as dodecyl-β-D-maltoside, though the tails have been truncated, as we cannot distinguish the two detergents in the crystallization screen. In the final cycles of refinement all data to 3.7 Å were used rather than the anisotropy-corrected data.

Superimpositions were carried out with LSQMAN^49^ such that all matching Cα pairs were <3.8 Å apart after superposition. The solvent accessible areas as well as the dimer interface area were calculated using AREAIMOL in the CCP4 suite^36^. A summary of the crystallographic data collection and phasing statistics is given in Supplementary Table 1. All crystallographic structure images were prepared using PyMOL^50^.

### Generation of an outward-facing UapA model

The human AE1 comprises 14 transmembrane helices classified into gate and core domains. The structure of AE1 was obtained in the outward-facing conformation and revealed that the two gate domains form a dimer interface. TMs 5 and 12 both cover the substrate-binding site in AE1 and are part of the dimerization domain. UapA was crystallized in the inward open state. By superimposing the core domains of UapA and AE1, a rigid movement of the core domains from the inward open to the outward open states can be inferred. Superimposing both the gate domains of the UapA dimer onto the gate domain of AE1 allowed us to postulate an outward open conformation of UapA.

### *A. nidulans* strains and growth conditions

Standard complete and minimal media for *A. nidulans* were used. Media and supplemented auxotrophies were at the concentrations given in the Fungal Genetics Stock Center database (http://www.fgsc.net). Nitrogen sources were used at final concentrations of 10 mM ammonium tartrate or 0.5 mM uric acid or other purines. The recipient for transformations was the *uapA*Δ *uapC*Δ *azgA*Δ *pabaA1 argB2* mutant strain lacking the endogenous *uapA* gene, as well as the genes encoding a secondary xanthine/uric acid transporter (UapC) and the major purine transporter AzgA. Plasmids carrying *gfp*-tagged WT or mutant versions of *uapA* were transformed and strains selected on the basis of the *argB*^−^ (*argB2*) arginine auxotrophy complementation^51^.

### Construction of UapA mutants

Plasmids expressing mutant UapA-GFP versions (Q408E, Q408P, N409D and G411V) used for the dominant negative analysis (Fig. 4) were constructed previously^19^. For the present work, the following UapA mutations were also made and functionally analysed: F155G, T404A, G411L, L459A, Q408E/A469E, Q408E/R481G/T526M and Q408E/R481G/F528S. All *uapA* mutations appearing in this work were generated using specific oligonucleotides (details provided in Supplementary Table 2), the QuikChange Mutagenesis Kit and plasmid pAN510exp-GFP carrying a *gfp*-tagged *uapA* gene, as a template^7^,^51^. For construction of the double and triple mutants the Q408E mutation was introduced by site-directed mutagenesis into existing vectors carrying the Q113L, A469E, T526M, R481G/T526M and R481G/F528S mutations^7^,^8^. Mutations F406Y, A441V, V463I, A469E and R481G were obtained previously as suppressors of mutation F528S. The single mutations were separated from F528S by replacing the SacI/SacI fragment from each double mutant with the corresponding fragment of pAN510exp-GFP^8^. Q113L/F528S and R481G/T526M were constructed previously by replacing the ClaI/XbaI fragment of pAN510exp-UapA-Q113L or the SacI/SacI fragment of pAN510exp-UapA-T526M with the corresponding fragments from pAN510exp-UapA-F528S or pAN510exp-UapA-R481G, respectively^8^. GFP C-terminal tagging has previously shown to have absolutely no effect on UapA transporter kinetics^51^. Mutant alleles of *uapA* were introduced by genetic transformation^52^. Transformants selected on arginine auxotrophy complementation were purified and those arising from single-copy plasmid integration, as evidenced by PCR and Southern blot analysis, identified. Selected transformants were tested for their ability to grow on uric acid or xanthine (major UapA substrates) or other purines at 25 or 37 °C, at pH 6.8, and for their subcellular localization, using epifluorescence inverted microscopy (Zeiss Observer Z1/AxioCam HR R3 camera/Zen lite 2012 software). Samples for standard fluorescence microscopy were prepared as previously described^53^. Strains co-expressing from the native *uapA* promoter WT UapA (that is, from the endogenous genetic locus^53^) and UapA-Q408P, UapA-Q408E, UapA-G411V or UapA-N409D (from single-copy plasmid integrations in the *argB* locus^19^), were constructed by standard genetic crossing of two parental strains, one expressing the WT gene and the other expressing the mutant allele. Previous experiments have shown that UapA steady-state levels arising from expression from its endogenous genetic locus, or from single-copy plasmid integration in the *argB* locus, are very similar. Consequently, the transport rate in the strain expressing two copies of WT UapA is nearly double (∼185%). In a negative control strain carrying a deletion of the *uapA* gene (*uapA*Δ), xanthine uptake is insignificant (<10%), because the genetic background used, in all cases, also lacks all other transporters specific for purines. Mutations Q408P, Q408E, G411V and N409D lead to UapA versions that are normally localized in the plasma membrane, but show no or very low (5–10%) transport activity, under standard uptake conditions.

### Transport assays

UapA mutants were functionally characterized by uptake assays performed in living cells. Radiolabelled [^3^H]-xanthine (22.8 Ci mmol^−1^, Moravek Biochemicals, CA, USA) uptake measurements were performed with *A. nidulans* germinating conidiospores concentrated at 10^7^ conidiospores per 100 μl at 37 °C, pH 6.8, at a defined developmental stage, in which UapA and other transporters show maximal expression, as previously described^54^. Competition experiments of [^3^H]-xanthine uptake were carried out with increasing concentrations of unlabelled hypoxanthine (1–1,000 μΜ). All transport assays were carried out in at least three independent experiments, with three replicates for each concentration or time point and s.d. was <20 % in all cases.

### Molecular dynamics

The protein was prepared for molecular simulation studies using the Protein Preparation Workflow (Schrödinger Suite 2015 Protein Preparation Wizard) implemented in the Schödinger suite and accessible from within the Maestro programme (Maestro, version 10, Schrödinger, LLC, New York, NY, 2015). Briefly, the hydrogen atoms were added and the orientation of hydroxyl groups, Asn, Gln, and the protonation state of His were optimized to maximize hydrogen bonding. Finally, the ligand–protein complex was refined with a restrained minimization performed by the Impref utility, based on the Impact molecular mechanics engine (Impact version 6.6, Schrödinger, LLC, New York, NY, 2015) and the OPLS2001 force field, setting a maximum root mean squared difference of 0.30. Ligand preparation for docking was performed with the LigPrep (LigPrep, version 2.5, Schrödinger, LLC, New York, NY, 2011) application that consists of a series of steps that perform conversions, apply corrections to the structure, generate ionization states and tautomers, and optimize the geometries. The homodimeric WT UapA crystal structure with xanthine present in the substrate-binding site of both monomers was utilized as the initial input structure. For the MD simulations Desmond v.3 software was applied (Desmond Molecular Dynamics System, version 3.8, D. E. Shaw Research, New York, NY, USA, 2008)^55^. The system was prepared by embedding the protein in a POPC lipid bilayer, solvating the membrane by TIP3P explicit water, neutralizing with counter ions and adding 150 mM salt. The stepwise equilibration protocol for membrane proteins as developed by Desmond has been followed on eight steps. No driving forces were applied to the ligand. The atomic Cα root mean squared difference of the two monomeric units following equilibration was 1.05 Å. Constant pressure and constant temperature (NPT) runs were performed for 30 ns simulations in three independent experiments with Langevin thermostat and barostat and semi isotropic pressure restraints for the substrate studied (xanthine). Root mean squared fluctuation (RMSF) calculations were based on frame 0 as a reference while Cα atoms of all residues were selected for RMSF calculation and fitting. Whole-residue RMSF ranged from 1 Å for rigid secondary structural elements to 6 Å for mobile loops in both monomers similar to what has been observed for membrane proteins. Figures were created with Maestro v10.1 (Schrödinger, LLC). All trajectory analysis was performed using the Simulation Event Analysis as implemented in Maestro version 10.

### Preparation and analysis of the 8-bromoxanthine

Bromine (3.29 mmol) was added to a solution of xanthine (1.31 mmol) in water (1.3 ml). The resultant mixture was refluxed for 2 h. After the solution had cooled to room temperature, the product was isolated by filtration. The product was washed sequentially with water (2 × 10 ml) and diethyl ether (2 × 10 ml) and subsequently dried *in vacuo* to give 8-bromo-1*H*-purine-2,6(3*H*,7*H*)-dione, abbreviated to 8-bromoxanthine in the manuscript. The final yield of material was 0.64 mmol, a yield of 49%. ^1^H NMR analysis was used to confirm the absence of trace xanthine impurity in the product. The pure 8-bromoxanthine was used for co-crystallization, fungal growth assays and transporter uptake assays (as detailed above). Where 8-bromoxanthine was used, this replaced xanthine during all the steps of purification of UapAG411VΔ_1–11_ and crystals were obtained in the same conditions as with xanthine, both as described above. Data were collected at a wavelength of 0.9 Å and subjected to processing as described above with the non-derivatized xanthine.

## Additional information

**Accession codes:** The coordinates of the structure have been deposited in the Protein Data Bank under the accession code PDB 5I6C .

**How to cite this article:** Alguel, Y. *et al.* Structure of eukaryotic purine/H^+^ symporter UapA suggests a role for homodimerization in transport activity. *Nat. Commun.* 7:11336 doi: 10.1038/ncomms11336 (2016).

## Supplementary Material

Supplementary Information Supplementary Figures 1-11, Supplementary Table 1 and Supplementary References.
