# Supplementary material for: Structure of eukaryotic purine/H+ symporter UapA suggests a role for homodimerization in transport activity
Source: Nat Commun. 2016 Apr 18;7:11336. doi: 10.1038/ncomms11336 (PMC4837479; doi:10.1038/ncomms11336)
Supplement: Supplementary Information — Supplementary Figures 1-11, Supplementary Table 1 and Supplementary References. [file ncomms11336-s1.pdf]

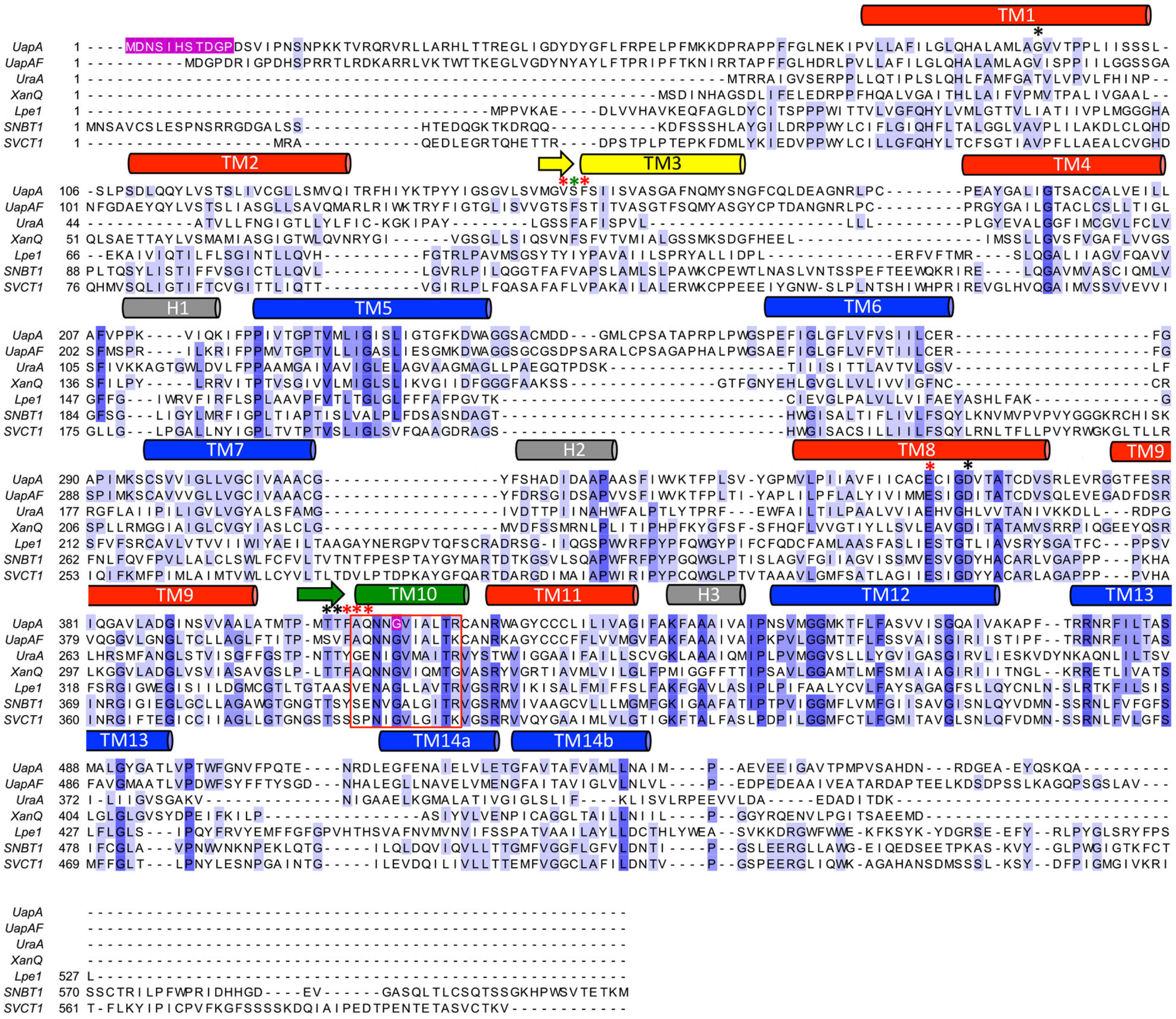

Supplementary Figure 1 Sequence alignment of UapA with selected NAT family members. UapA (Q07307) from *A. nidulans*; UapAF (XP748919), a uric acid/xanthine transporter from the pathogenic fungus *A. fumigatus*; UraA (YP490725) a uracil transporter from *E. coli;* XanQ (P67444) a xanthine transporter from *E. coli*; Lpe1 (U43034) a uric acid/xanthine transporter from maize; SNBT1 (AB511909) a uracil transporter from rat; SVCT1 (BC050261) a human L-ascorbate transporter. The selected sequences were aligned using Clustal Omega[1] and the figure edited in JalView[2]. The secondary structure elements of UapA are indicated by the cylinders and arrows located above the sequence and coloured as seen in the structure images in Figure 1. The regions of UapA modified in order to obtain a stable protein for structural studies are coloured in magenta. The NAT motif is indicated by the red box. Residues involved in direct binding of xanthine are indicated by the red asterisks, other residues contributing to the architecture of the binding site are indicated by the black asterisks and Ser154 with a role in substrate specific binding is indicated by the green asterisk.

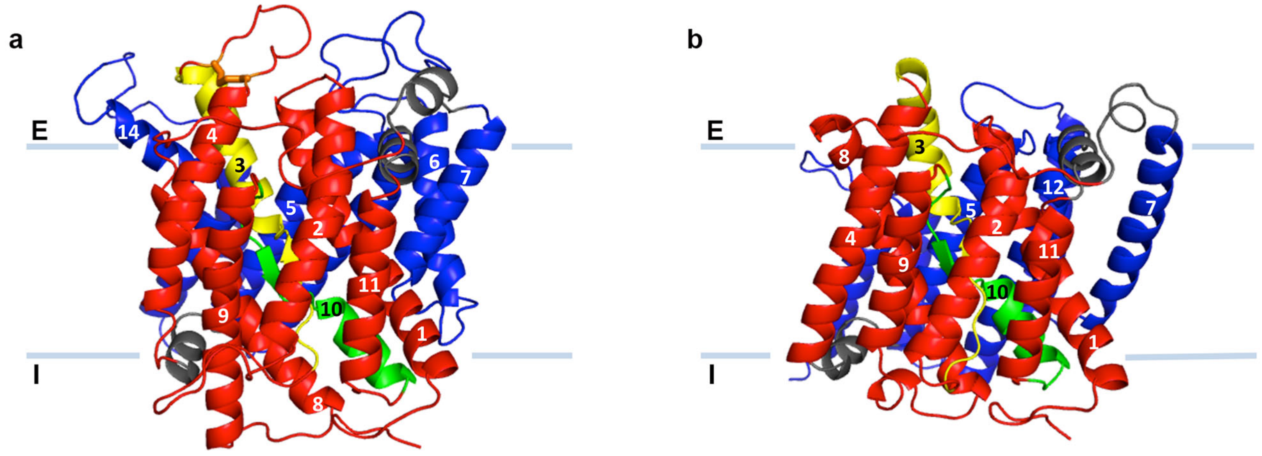

Supplementary Figure 2 Comparison of UapA and UraA. Ribbon representations of monomers of both **a,** UapA and **b,** UraA b, PDB 3QEZ), coloured as in Figure 1. The proteins are shown looking through the membrane from the core domain. The pale blue lines indicate the likely location of the membrane with the extracellular (E) and intracellular (I) sides of the membrane labeled. The comparison clearly shows that UraA is a more compact protein with much shorter loop regions than UapA.

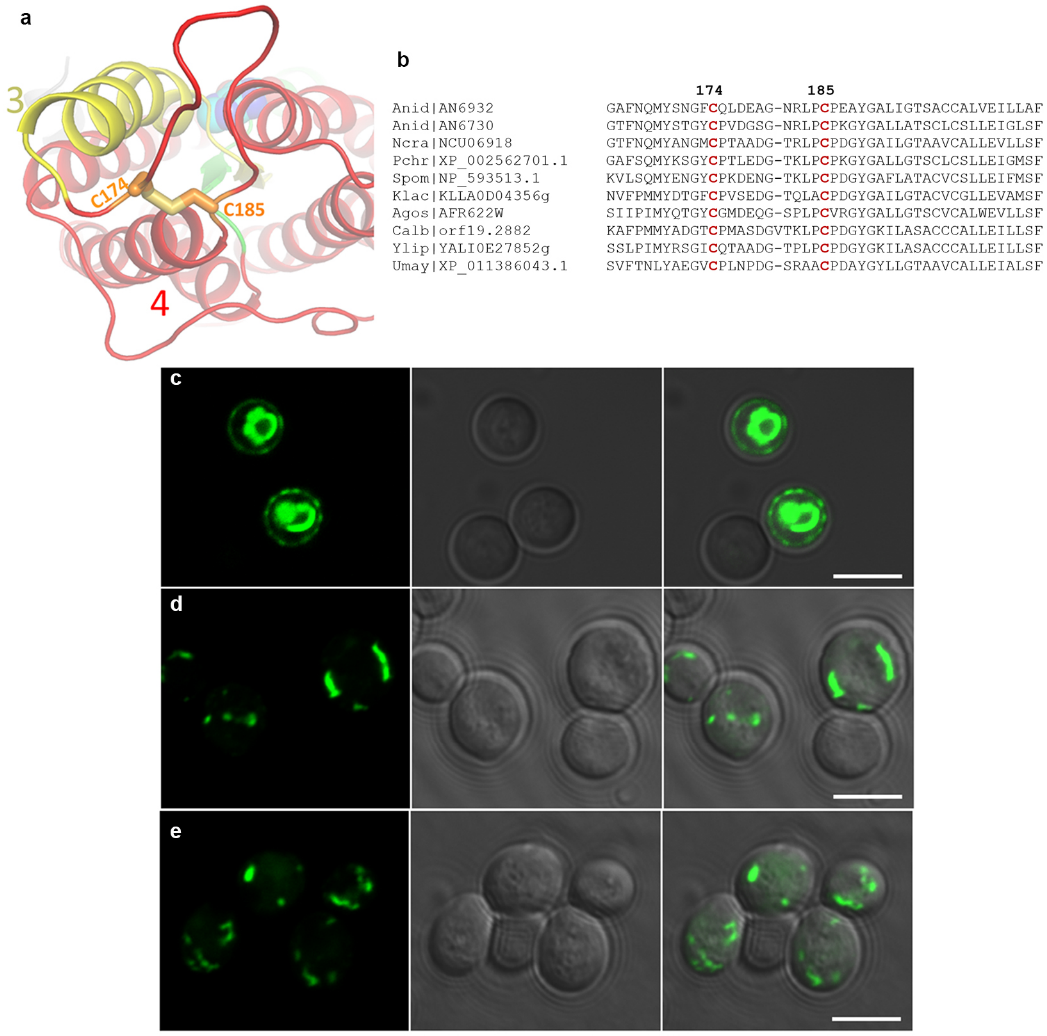

Supplementary Figure 3 The UapA disulphide bridge a, Zoomed in region of the disulphide bond of UapA. The protein is shown from the extracellular side in ribbon representation and coloured as shown in Figure 1. Cys174 and Cys185, indicated in orange sticks, are located in the extracellular loop between TMs 3 and 4 in the core domain. The disulphide bond is shown in pale yellow. **b,** Alignment of UapA (AN6932) with homologues from selected representatives of major taxonomic groups. AN6730 is UapC, a paralogue of UapA in *A. nidulans*. Ncra, *Neurospora crassa*; Pchr, *Penicillium chrysogenum*; Spom, *Schizosaccharomyces pombe*; Klac, *Kluyveromyces lactis*; Agos, *Ashbya gossypii;* Calb, *Candida albicans*; Ylip, *Yarrowia lipolytica*; Umay, *Ustilago maydis*. Cys174 and Cys185, indicated in red, are two of a total of 19 Cys residues in UapA. Mutants C174S and C185S were made in the UapAG411V$_{\Delta 1\text{-}11}$ background and expressed as GFP fusions in *S. cerevisiae*. Both mutants expressed to significantly lower levels (~0.8 mg/L) than the control protein, UapAG411V$_{\Delta 1\text{-}11}$, (~2.8 mg/L). Fluorescence microscopy images revealed that the **d,** C174S and **e,** C185S proteins failed to traffic to the membrane, unlike **c,** the control UapAG411V$_{\Delta 1\text{-}11}$, indicating incorrect folding of both Cys mutants. Data is representative of n = 3 independent experiments. Scale bar indicates 10 nm.

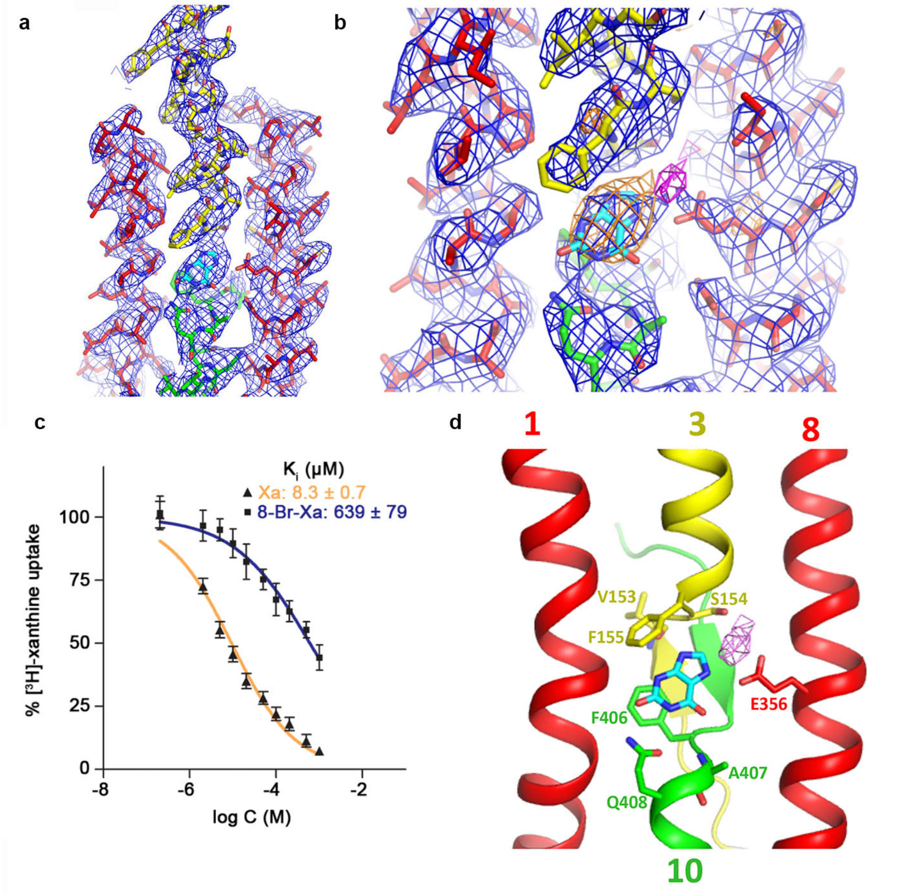

Supplementary Figure 4 Substrate binding in UapA. a, Electron density. The final $2mF_o$-DF_c electron density map, contoured at sigma level 1.0 showing xanthine in cyan binding in chain A. **b**, Illustration of a $2mF_o$-DF_c omit map in blue contoured at sigma level 1.0 and F_o-F_c omit map in brown contoured at sigma level 3.0 are overlapped with the anomalous peak for the bromine of 8-bromoxanthine contoured at sigma level 3.7 and shown in magenta. Xanthine is put into the omitted region as stick model with carbons coloured in cyan. **c,** The Ki of 8-bromoxanthine for wild-type UapA was estimated to be 639 ± 79 µM (± s.d., n = 9). Data shown is representative of n = 3 independent experiments. **d,** Zoomed in view of the UapA substrate binding site coloured as in Figure 1 with anomalous difference density calculated using the 8-bromoxanthine in magenta. The model was refined against the 8-bromoxanthine dataset processed to 4 Å resulting in a R-factor and R-free of 31.4% and 37.4%. The resulting anomalous map contoured at a sigma level of 3.7. The side chain groups of key residues involved in substrate binding are shown as a stick model and labeled. The position of the peak next to C8 of xanthine is consistent with the anomalous signal obtained for the 8-bromoxanthine and confirms the orientation of xanthine as predicted by mutagenesis studies[3]. In addition to xanthine, UapA also binds and transports uric acid. In uric acid, the bromine of the 8-bromoxanthine is replaced by an oxygen atom. Previous mutational analysis highlighted Ser154 as forming substrate specific interactions with uric acid[4]. The location of the bromine atom supports Ser154 being involved in substrate specific binding of uric acid but not xanthine.

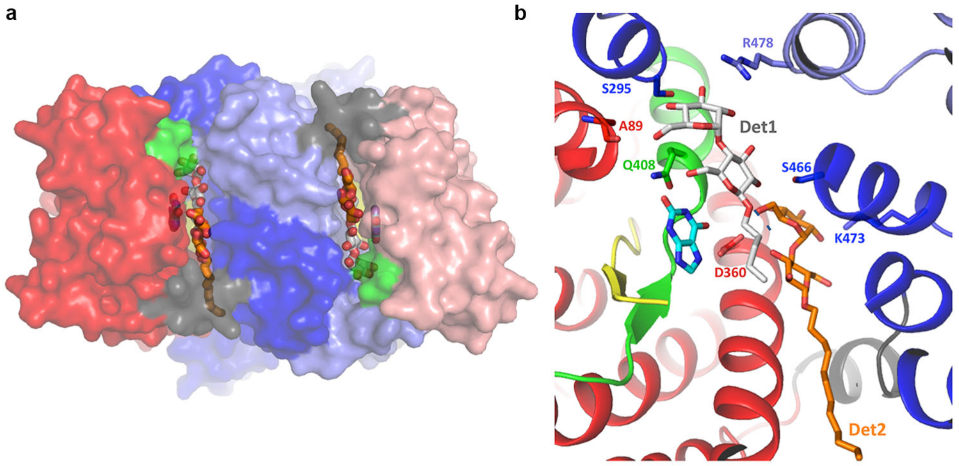

Supplementary Figure 5 Detergent binding to UapA a, The UapA dimer is shown as a surface representation from the cytoplasmic side of the protein with both monomers coloured as in Figure 1. Xanthine is shown in cyan space filling model and the detergents in white and orange stick model. **b,** Zoomed in view showing the likely interactions between the protein and substrate and the bound detergent molecules. The protein regions are shown in ribbon representation and coloured as in Figure 1. Xanthine, Detergent 1 and Detergent 2 are all shown in stick representation in cyan, white and orange respectively. Amino acid residues likely to form interactions with the detergent molecules are shown in stick model and labelled. The xanthine is fully accessible to the inward-facing side of the protein through this detergent-filled cavity.

a

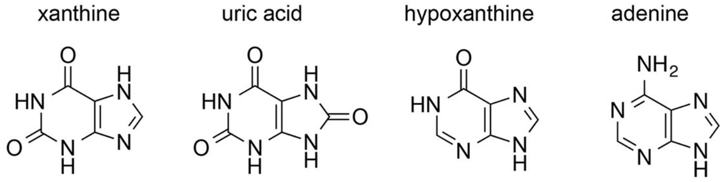

b

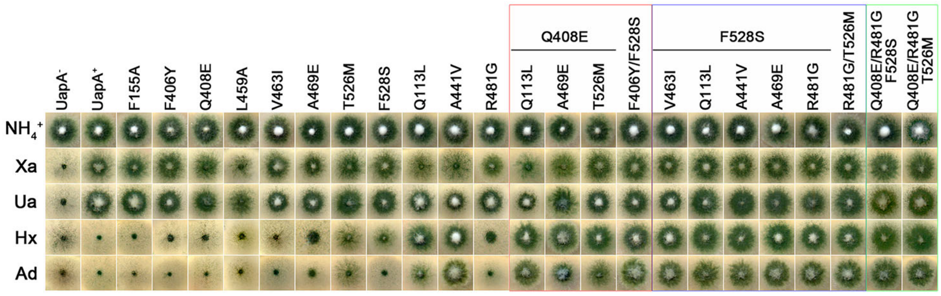

c

alleles	Location+	Ki (μM)			
		Xa	Ua	Hx	Ad
**wt**		8 ± 0.6	7 ± 0.5	>2000	>2000
**F155A**	TM3, BS	10 ± 1.9	17 ± 3.5	905 ± 142	1035 ± 154
**F406Y***	TM10, BS	3 ± 0.9	11 ± 2.4	>2000	>2000
**Q408E****	TM10, BS	16 ± 3.1	21 ± 4.1	71 ± 6.3	>1000
**L459A**	TM12, I	19 ± 2.6	9 ± 1.6	760 ± 109	738 ± 94
**A469E***	TM12, I	8 ± 0.5	58 ± 6.8	880 ± 84	>1000
**R481G***	TM13, OM	18 ± 3.8	19 ± 3.9	>2000	>2000
**F528S***	TM14, I	4 ± 0.5	10 ± 1.8	>2000	>2000
**T526M****	TM14,I	5 ± 0.6	12 ± 1.7	>1000	>2000
**R481G/T526M***		2 ± 0.6	8 ± 0.8	179 ± 19	980 ± 110
**Q408E/T526M****		28 ± 4.1	7 ± 0.9	16 ± 3.2	950 ± 134
**Q408E/A469E**		14 ± 3.1	16 ± 2.4	370 ± 44	>2000
**Q408E/R481G/F528S**		21 ± 3.8	64 ± 7.5	145 ± 12.5	188 ± 16.3
**Q408E/R481G/T526M**		9 ± 1.2	39 ± 5.3	24 ± 4.5	83 ± 7.5

+Location of the residue; BS = Binding site, I = Interface between the gate and core domains and OM = Opposite monomer.

Supplementary Figure 6 Combining mutations from different specificity regions expands the substrate profile of UapA. a, Structures of substrates used in growth tests. Hypoxanthine and adenine do not bind to wild-type UapA. **b,** Growth tests of isogenic *A. nidulans* strains containing wild-type and mutant versions of UapA on either ammonium (NH_4^+), xanthine (Xa), uric acid (Ua), hypoxanthine (Hx) or adenine (Ad) as sole nitrogen sources. $UapA^-$ indicates an isogenic strain carrying a total deletion of the *uapA* gene. $UapA^+$ indicates a strain expressing wild-type UapA from its native endogenous locus. All strains shown also carry genetic deletions of two other major purine transporters (*azgA* and *uapC*[3]). **c,** Inhibition constants (Ki) for binding of Xa, Ua, Hx and Ad by UapA mutants in competition with [^{3}H] xanthine are shown ± s.d., n = 9. Data for some mutants are taken from *[5] and **[6] and are shown for comparison. Single mutants, which expand the substrate profile of UapA, were identified by unbiased genetic screening through direct selection of UapA-mediated growth on hypoxanthine or adenine[4-6]. For clarity just a single substrate concentration (0.5 mM) was used in the fungal growth experiments. Notice that most of the single mutants do not allow significant UapA-mediated growth when Hx or Ad is provided at this concentration. Of the single mutants only Q113L and A441V support some growth on 0.5 mM Hx or Ad. The F406Y and Q408E mutants allow binding of Hx and Ad at high (>2 mM) concentration[5] but do not transport these non-native substrates. Importantly, F406Y or Q408E mutations in the binding site, in combination with mutations from other specificity regions confer full growth at 0.5 mM Hx and Ad. Data is representative of n = 3 independent experiments.

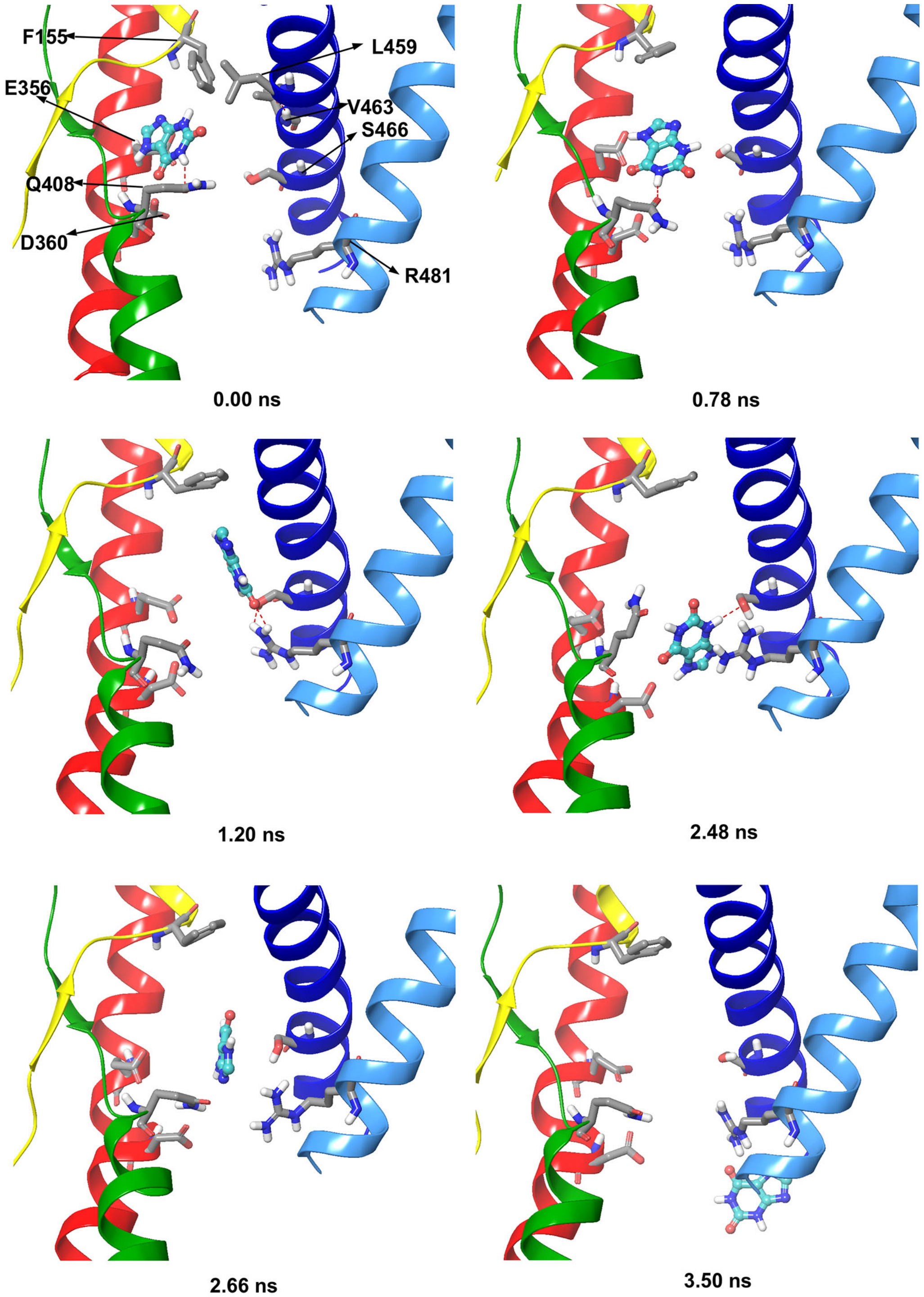

Supplementary Figure 7 Stepwise transport of xanthine across the UapA inward-facing structure as extracted from MD simulations. Representative structures at characteristic time points as revealed from MD analysis (see also **Supplementary Figure 8**). Xanthine, shown in cyan coloured stick model, interacts with residues characterised as crucial for transporter activity forming hydrophobic, π-π or H-bond interactions (red dashed lines) in the binding cavity. Movement of the substrate is closely associated with reorientation of the side chain of Arg481 from the opposite monomer which protrudes into the binding cavity and mediates the sliding movement of the substrate towards the cytoplasmic side of the transporter. Note the outward movement of TM10 (green helix) away from the translocation channel as the substrate moves through. Data is representative of n = 3 independent experiments.

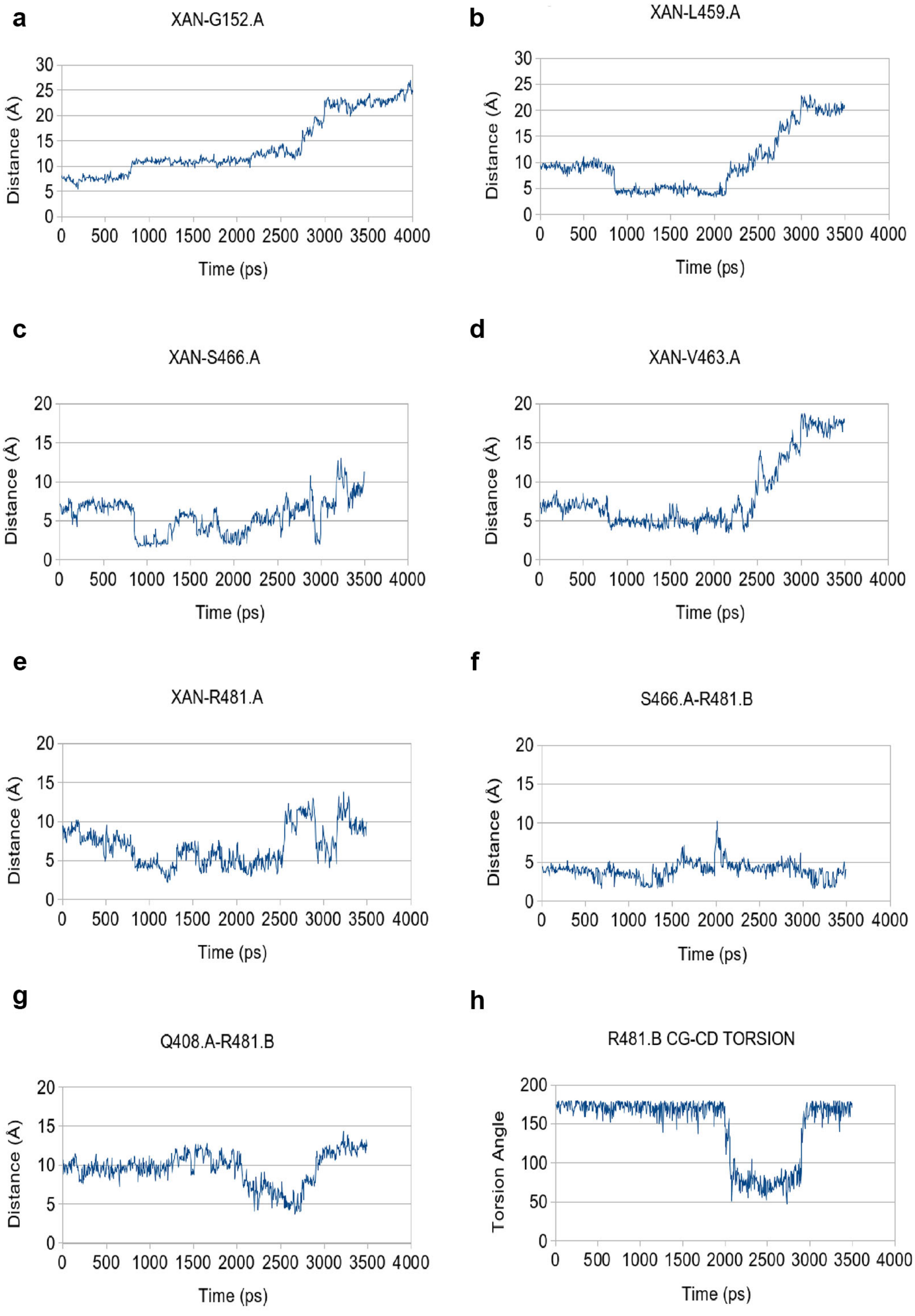

Supplementary Figure 8 Atomic distance fluctuation plots between xanthine and crucial residues, and residue conformational changes during MD calculations. a, The translocation pathway of xanthine was followed showing the distance of the ligand from Gly152 (the residue exhibiting the lowest RMSF). **b, c and d,** Xanthine is moving towards Leu459, Ser466 and Val463, while Arg481 from the opposite monomer is approaching the central binding cavity. **e and f,** Arg481 forms H-bonds with both Ser466 and xanthine. **g,** A conformational change of the Arg481 side chain from the opposite monomer facilitates xanthine translocation, while **h,** the distance between Arg481 and Gln408 reaches a minimum. All distances are referring to specific atoms (in bold) from xanthine and residues of monomer A except Arg481 from monomer B. **a**, **C5**(xanthine) – **Cα**(G152), **b**, **N9**(xanthine) – **Cγ**(Leu459), **c**, **N3**(xanthine) – **O**H(Ser466), **d**, **N9**(xanthine) – **Cδ**(Val463), **e**, **O2**(xanthine) - Nη**H**(Arg481), **f**, **O**H(Ser466) – Nη**H**(Arg481), **g**, C=**O**(Asn408) - Nη**H**(Arg481), **h**, conformational changes of Arg481 Cγ-Cδ bond torsional angle.

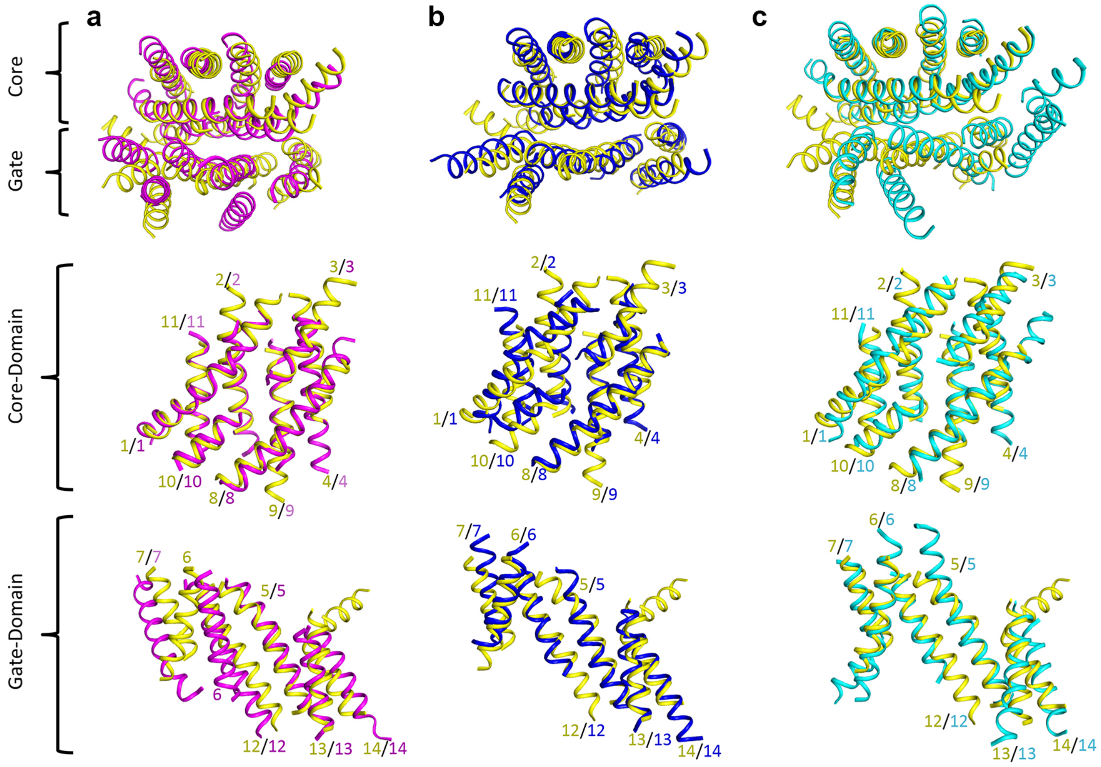

Supplementary Figure 9 Comparison of UapA with other related transporter structures. Superposition of UapA (yellow) with **a,** UraA (magenta; PDB 3QE7), **b,** SLC26Dg (blue; PDB 5DAO) and **c,** the human AE1 transporter (cyan; PDB 4YZF). In the top panel the complete monomers have been superposed based on their respective core domains, in the middle panels the superposed core domains are shown and in the lower panel the gate domains. The gate domain of UraA differs markedly from the other three structures. This may reflect the true structure of UraA, but it is also possible that the detergent molecule located between these helices has forced the protein to adopt a non-physiological conformation. The key difference in conformational state between the inward and outward facing transporter forms is highlighted by the superposition of the AE1 (outward facing) and UapA (inward facing).

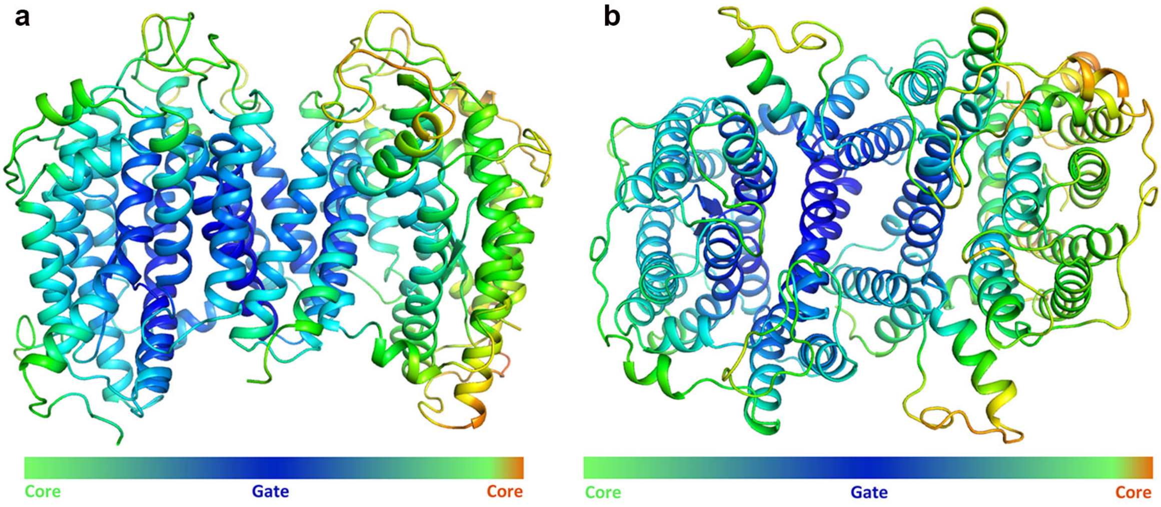

Supplementary Figure 10 Flexibility of the UapA dimer. Ribbon diagram of the UapA dimer coloured by B-factor with dark blue indicating the least flexible regions and orange indicating the most flexible regions. The core domain is clearly more flexible than the gate domain. The protein is shown both **a,** looking through the membrane and **b,** from the intracellular side of the protein.

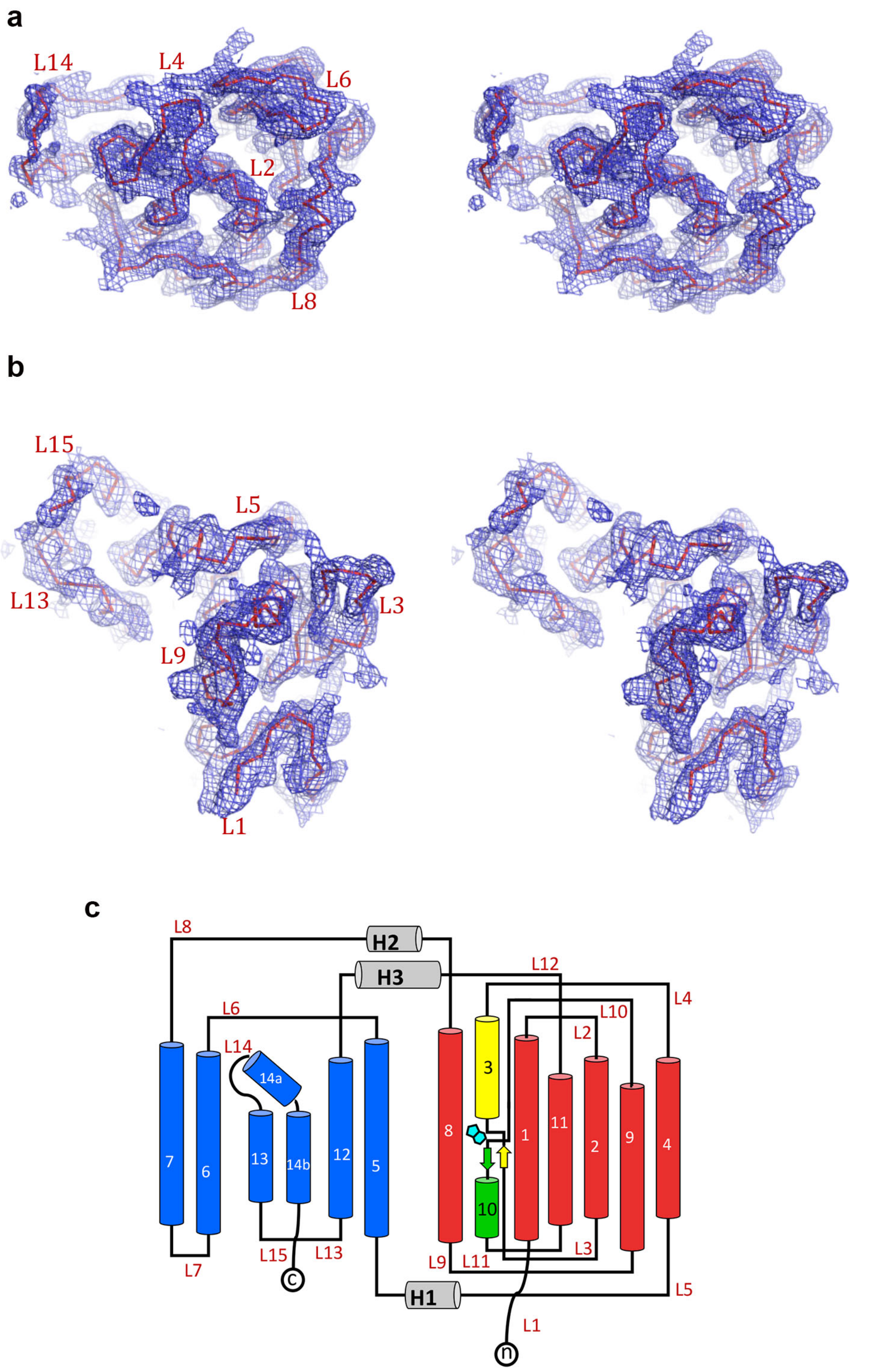

Supplementary Figure 11 Electron density and fitting of the loops of UapA. A 2mF$_o$-DF$_c$ composite omit electron density map was calculated, and views are shown here in stereo contoured at sigma level 1.0 of both the intracellular **a**, and extracellular **b,** loops. The loops are labeled in accordance with the **c,** topology. The electron density map is very good quality for the resolution. We were therefore able to build a single polypeptide chain including all the loops, which corresponded to almost continuous density.

**Supplementary Table 1** Data collection and phasing statistics				
	**UapAG411V$_{\Delta 1\text{-}11}$**	**UapAG411V$_{\Delta 1\text{-}11}$ - TaBr**	**Isomorph UapAG411V$_{\Delta 1\text{-}11}$**	**8-BromoXanthine**
Beamline	I04-DLS	I03-DLS	I03-DLS	I02-DLS
Space Group	*P2₁*	*P2₁*	*P2₁*	*P2₁*
Resolution (Å)[a]	76.9 -3.7 (3.8-3.7)	80.6-7.7 (7.86-7.7)	89.6-5.8 (5.90-5.8)	39.12-4.0 (4.47-4.0)
Cell dimensions a,b,c, (Å) α, β, γ (°)	75.1, 173.8, 82.4 90, 111.1, 90	76.1, 178.5, 84.6 90,107.8, 90.	76.3, 179.1, 84.1 90, 107.8, 90	75.3, 176.8, 82.0 90, 110.2, 90
Wavelength (Å)	0.992	1.25477	1.25807	0.9
Number of measured reflections	51869	16842	20510	99487
Number of unique reflections	20599	2599	6091	16993
Completeness (%)	97.8 (97.7)	98.6 (100)	98.3 (98.8)	99.6 (99.8)
Redundancy	2.5 (2.5)	6.5 (6.8)	3.4 (3.3)	5.9 (6.0)
I/σ(I)	7.7(1.0)	12.9 (4.1)	15.4 (1.8)	6.0 (1.5)
R_{merge} (%)[b]	10.2(96.5)	6.6 (39.3)	2.9 (63.2)	10.7 (1.093)
$CC_{0.5}$	0.99(0.51)	0.99 (0.94)	0.99 (0.79)	0.99 (0.73)
R_{cullis} [c]		0.539		
Phasing Power [d] isomorphous Centric Acentric		1.9 1.5		
Phasing Power anomalous		1.0		

**Refinement statistics**	
R_{factor}[e] (%)	29.6
R_{free}[f] (%)	32.7
R.m.s.d. from ideal values	
R.m.s. bond lengths (Å)	0.006
R.m.s. bond angles (°)	1.103
Ramachandran plot	
outliers [g] (%)	1.7
favoured (%)	98.3
Average B-values (Å^2)	
Uapa: A;B;	164.4
Xanthine	141.6
Detergent	159.8

[a] Values in parentheses refer to data in the highest resolution shell.
[b] $R_{merge} = \sum_{hkl}\sum_i |< I(hkl) > - I(hkl)_i| / \sum_{hkl}\sum_i I(hkl)_i$
[c] $R_{cullis} = \Sigma\big|F_{PH}-|F_P+F_H|\big| / \Sigma|F_{PH}-F_P|$
[d] Phasing power = rms $(|F_H| / \big|F_{PH}-|F_P+F_H|\big|)$
[e] $R_{factor} = S|F_{obs}-F_{calc}| / S\ F_{obs}$.
[f] The R_{free} is the same as the R_{factor} but for the 5% of test reflections.
[g] as defined in MolProbity[8].

**Supplementary Table 2** **Oligonucleotide sequences**		
	**Oligonucleotide sequence (5'-3')**	**Reference**
**UapA$\Delta_{1\text{-}11}$**		
Forward	TCGACGGATTCTAGAACTAGTGGATCCCCCATGGACTCCGTCATCCCCAA	[7]
Reverse	AAATTGACCTTGAAAATATAAATTTTCCCCAGCCTGCTTGCTCTGATCTCC	[7]
**Mutation**		
F155A	CTCAGTTATGGGGGTCTCGGCCTCCATCATCTCCGTCGCC	[4]
F155G	CTCAGTTATGGGGGTCTCGGGATCCATCATCTCCGTCGCC	this study
C174S	TACTCGAACGGGTTCAGTCAACTCGACGAGG	this study
C185S	GGCTGGAAACAGACTCCCTAGCCCCGAAGC	this study
T404A	GCGACAATGACCCCCATGGCGACCTTTGCGCAGAACAAC	this study
Q408E	CCATGACGACCTTTGCGGAGAACAACGGCGTGATTGC	[3]
Q408P	CCATGACGACCTTTGCGCCGAACAACGGCGTGATTGC	[3]
N409D	GACGACCTTTGCGCAGGACAACGGCGTGATTGCCC	[3]
G411V	TTGCGCAGAACAACGTCGTGATTGCCCTCAC	[3]
G411L	TTGCGCAGAACAACCTGGTGATTGCCCTCAC	this study
L459A	GGGCGGGATGAAGACGTTTGCCTTCGCTTCGGTCGTTATTAG	this study

References for Supplementary data

1. Sievers, F. *et al*. Fast, scalable generation of high-quality protein multiple sequence alignments using Clustal Omega. *Molecular Systems Biology* **7,** 539–539 (2011).
2. Waterhouse, A. M., Procter, J. B., Martin, D. M. A., Clamp, M. & Barton, G. J. Jalview Version 2--a multiple sequence alignment editor and analysis workbench. *Bioinformatics* **25,** 1189–1191 (2009).
3. Koukaki, M. *et al*. The Nucleobase-ascorbate Transporter (NAT) Signature Motif in UapA Defines the Function of the Purine Translocation Pathway. *Journal of Molecular Biology* **350,** 499–513 (2005).
4. Amillis, S., Kosti, V., Pantazopoulou, A., Mikros, E. & Diallinas, G. Mutational Analysis and Modeling Reveal Functionally Critical Residues in Transmembrane Segments 1 and 3 of the UapA Transporter. *Journal of Molecular Biology* **411,** 567–580 (2011).
5. Kosti, V., Papageorgiou, I. & Diallinas, G. Dynamic Elements at Both Cytoplasmically and Extracellularly Facing Sides of the UapA Transporter Selectively Control the Accessibility of Substrates to Their Translocation Pathway. *Journal of Molecular Biology* **397,** 1132–1143 (2010).
6. Papageorgiou, I. *et al*. Specific Interdomain Synergy in the UapA Transporter Determines Its Unique Specificity for Uric Acid among NAT Carriers. *Journal of Molecular Biology* **382,** 1121–1135 (2008).
7. Leung, J., Cameron, A. D., Diallinas, G. & Byrne, B. Stabilizing the heterologously expressed uric acid-xanthine transporter UapA from the lower eukaryote *Aspergillus nidulans*. *Mol. Membr. Biol.* **30,** 32–42 (2013).
8. Chen, V. B. *et al*. MolProbity: all-atom structure validation for macromolecular crystallography. *Acta Crystallogr. D Biol. Crystallogr.* **66,** 12–21 (2010).
